# Plastomes of eight *Ligusticum* species: characterization, genome evolution, and phylogenetic relationships

**DOI:** 10.1186/s12870-020-02696-7

**Published:** 2020-11-13

**Authors:** Ting Ren, Zi-Xuan Li, Deng-Feng Xie, Ling-Jian Gui, Chang Peng, Jun Wen, Xing-Jin He

**Affiliations:** Key Laboratory of Bio-Resources and Eco-Environment of Ministry of Education, College of Life Sciences, Sichuan University, Chengdu, 610065 China

**Keywords:** *Ligusticum*, Plastome, Characterization, Relaxed selection, Evolution, Phylogenetic relationships

## Abstract

**Background:**

The genus *Ligusticum* consists of approximately 60 species distributed in the Northern Hemisphere. It is one of the most taxonomically difficult taxa within Apiaceae, largely due to the varied morphological characteristics. To investigate the plastome evolution and phylogenetic relationships of *Ligusticum*, we determined the complete plastome sequences of eight *Ligusticum* species using a de novo assembly approach.

**Results:**

Through a comprehensive comparative analysis, we found that the eight plastomes were similar in terms of repeat sequence, SSR, codon usage, and RNA editing site. However, compared with the other seven species, *L. delavayi* exhibited striking differences in genome size, gene number, IR/SC borders, and sequence identity. Most of the genes remained under the purifying selection, whereas four genes showed relaxed selection, namely *ccsA*, *rpoA*, *ycf1*, and *ycf2*. Non-monophyly of *Ligusticum* species was inferred from the plastomes and internal transcribed spacer (ITS) sequences phylogenetic analyses.

**Conclusion:**

The plastome tree and ITS tree produced incongruent tree topologies, which may be attributed to the hybridization and incomplete lineage sorting. Our study highlighted the advantage of plastome with mass informative sites in resolving phylogenetic relationships. Moreover, combined with the previous studies, we considered that the current taxonomy system of *Ligusticum* needs to be improved and revised. In summary, our study provides new insights into the plastome evolution, phylogeny, and taxonomy of *Ligusticum* species.

**Supplementary information:**

**Supplementary information** accompanies this paper at 10.1186/s12870-020-02696-7.

## Background

The genus *Ligusticum*, belonging to the family Apiaceae with approximately 60 species, is distributed throughout Asia, Europe, and North America [1]. It has two distribution centers: one in the Himalayas, and the other in North America [2]. There are 40 species (35 endemics) of this genus in China, most of which are restricted to alpine regions [1].

*Ligusticum* is one of the most taxonomically difficult genera within Apiaceae, largely due to the varied morphological characteristics of flowers, leaves, bracteoles, and mericarps that make it difficult to distinguish from its neighbors [1, 3, 4]. So far, its phylogenetic relationships with nearby genera are not clear, such as *Ligusticopsis*, *Tilingia*, *Cnidium*, *Selinum*, *Hymenidium*, *Pachypleurum*, *Rupiphila*, and *Paraligusticum* [1], especially, merging *Tilingia* and *Ligusticopsis* into *Ligusticum* is still debatable [2]. The diagnostic characters of *Tilingia* are the distinct calyx teeth and the mericarp bearing a vitta in each furrow [5], which do not distinguish it from *Ligusticum*. Thus, *Tilingia* was transferred into *Ligusticum* [6, 7]. Leute [3] separated *Ligusticopsis* from *Ligusticum* based on the prominent calyx teeth. This treatment is not supported, for some *Ligusticum* species also have this characteristic [2]. Traditional methods to distinguish these species are based on their morphological characteristics, while many above-mentioned species always exhibit similar characteristics leading to extremely difficult species classification and generic delimitation [1, 3, 4]. Previously, a few molecular markers have been used to study the phylogeny of *Ligusticum*, such as nuclear ribosomal DNA internal transcribed spacer (ITS), plastid DNA *rpl16*, *rps16*, and *rpoC1* intron [4, 8–14], yet the DNA fragments fail to recognize *Ligusticum* as a monophyletic group. Among which, Downie et al. [12] identified five clades within *Ligusticum*, including *Acronema* Clade, *Conioselinum chinense* Clade, Pyramidoptereae, Selineae, and *Sinodielsia* Clade. Zhou et al. [4] subsequently divided the genus *Ligusticum* into six clades, and East-Asia (*Physospermopsis*) Clade was added. It can be seen that the genus *Ligusticum* is facing a big challenge of taxonomy and phylogeny. Therefore, more genomic resources are needed for reconstructing phylogenetic relationships and re-evaluating the generic limits of *Ligusticum*.

Additionally, many *Ligusticum* species are precious traditional herbs with excellent medicinal values. For example, the rhizomes and roots of *L. jeholense* or *L. sinense* are used as the traditional Chinese medicine named Gao-ben, which has been widely used to treat colds, headaches, trapped wind, and rheumatic arthralgia [15]. As a result, this herb has been conducted many studies on bioactive, chemical components, or pharmacology [16, 17]. Despite excellent medicinal value, genomic resources are lacking and species authentication is difficult. Thus, it is necessary to develop more DNA barcodes by a comparative plastome method for species authentication to assure medicinal quality.

Plastid is a key organelle for green plants, which participates in the photosynthetic process and provides essential energy for plants [18]. The plastid genome (plastome) is a double-stranded molecule of 115 to 165 kb in most plants [19]. Structural organization, gene arrangement, and gene content of plastome are relatively conserved. Typical plastome contains a large single copy (LSC) region of 82–90 kb, a small single copy (SSC) region of 15–20 kb, and two inverted repeats (IRs) regions of 22–25 kb [19]. It always encodes 110–130 distinct genes, including protein-coding gene(~ 80), transfer RNA (tRNA) gene (~ 30), and ribosomal RNA (rRNA) gene (4) [20]. Moreover, it is usually uniparental inheritance and has low nucleotide substitution rates [21]. For these reasons, the plastome has become useful a tool for plant phylogenetic studies at different taxonomic levels [22–25]. Currently, the plastid phylogenomics analysis of *Ligusticum* has not been reported. Meanwhile, the ongoing development of next-generation sequencing and bioinformatics technology makes it cheaper and faster to obtain the complete plastome sequence than ever before. Therefore, we prefer to use the plastomes to infer the phylogenetic relationships for *Ligusticum*.

Here, we newly sequenced eight plastomes of *Ligusticum* species. To obtain a comprehensive understanding of phylogenetic relationships, we also used nuclear ITS sequences to construct the phylogenetic tree. Our aims were to (1) infer the plastome evolution of *Ligusticum*; (2) provide more genomic resources for developing candidate DNA barcodes; (3) test if the plastomes increase resolution than traditional DNA markers; and (4) serve as a reference for subsequent phylogenomics studies of this genus. Overall, the complete plastomes reported here will promote plastome evolution, phylogeny, and taxonomy studies of *Ligusticum*.

## Results

### Characteristics of *Ligusticum* plastomes

**Fig. 1:**
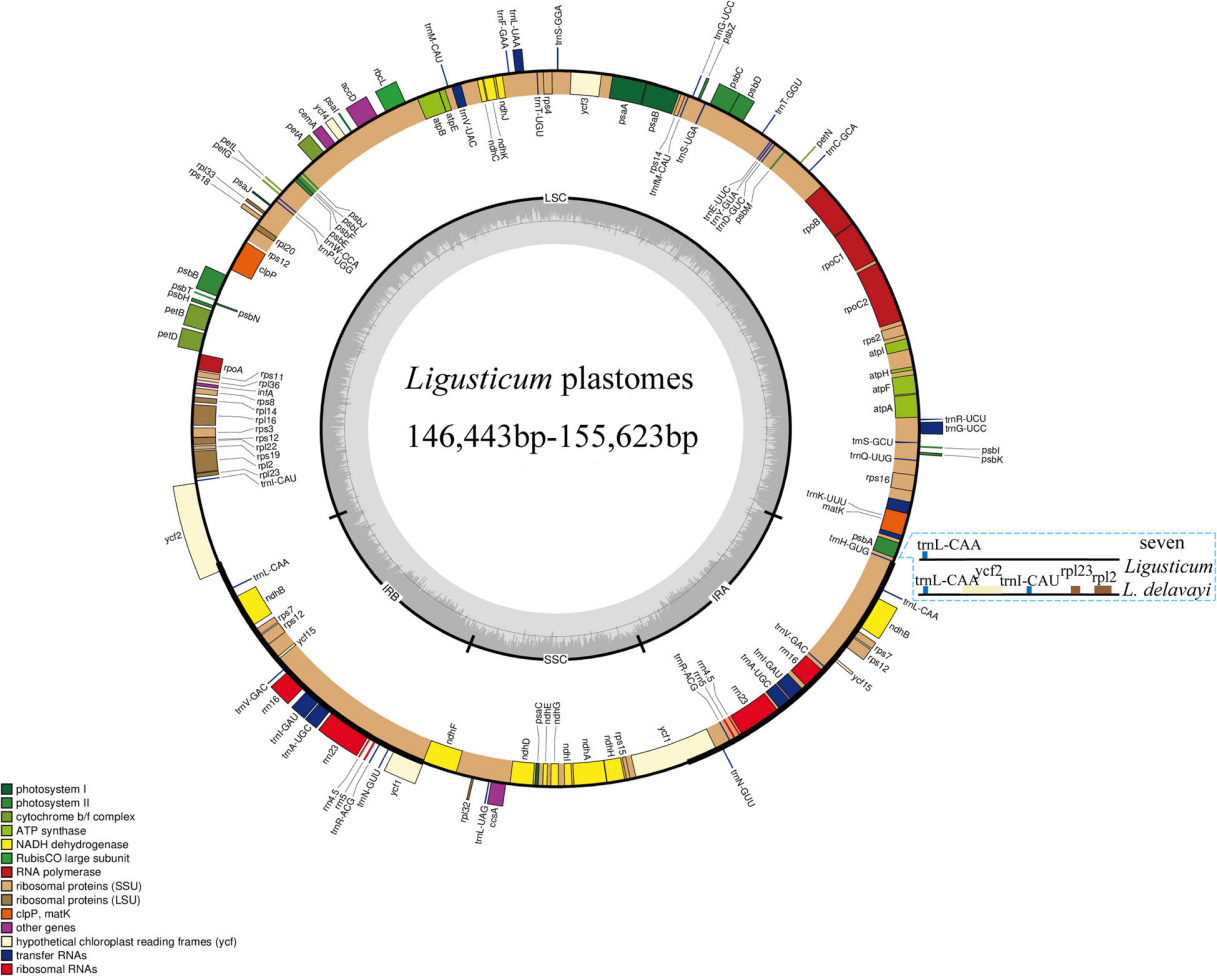
Gene map of eight *Ligusticum* plastomes. The genes shown outside of the circle are transcribed clockwise, while those inside are transcribed counterclockwise. The genes belonging to different functional groups are color-coded. The innermost darker gray represents the GC content of the plastome

After quality control, 5.76 Gb (*L. scapiforme*) to 7.47 Gb (*L. delavayi*) clean reads were generated for the eight *Ligusticum* species (Table 1), then we obtained eight complete plastome sequences by a de novo assembly. The determined complete plastome sequences of the eight *Ligusticum* species ranged from 146,443 bp in *L. pteridophyllum* to 155,623 bp in *L. delavayi* (Table 1). All of them were highly conserved in structure compared to most angiosperms, sharing the typical quadripartite structure with two copies of IR regions (18,166–26,908 bp), SSC regions (16,741–17,591 bp), and LSC regions (85,066–93,363 bp). The overall GC content was between 37.3–37.6%, while the IR regions were higher (42.5–44.8%) than that of the LSC (35.7–36.0%) and SSC (30.9–31.2%) regions (Table 1). The eight plastomes contained about 129–133 genes, including 85–88 protein-coding genes, 36–37 tRNA genes, and eight rRNA genes (Fig. 1, Table 1, Additional file 2: Table S1). *L. delavayi* contained four more genes (*ycf2*, *rpl23*, *rpl2*, and *trnI-CAU*) than seven other *Ligusticum* species in IRa (Fig. 1, Additional file 2: Table S1). The *rps12* gene was trans-spliced with the 5’end and the duplicated 3’end were located in the LSC and IR regions, respectively (Fig. 1). The *trnK-UUU* had the longest intron (2485–2543 bp) containing the *matK* gene (Fig. 1). The GC content of four rRNA (*rrn16*, *rrn23*, *rrn4.5*, and *rrn5*) genes was high (55.1–55.3%) (Fig. 1).

**Table 1:** Characteristics of the eight *Ligusticum* plastomes

	*L. capillaceum*	*L. delavayi*	*L. hispidum*	*L. involucratum*	*L. likiangense*	*L. pteridophyllum*	*L. scapiforme*	*L. thomsonii*
Raw reads (G)	6.17	7.52	6.5	7.24	5.87	7.21	5.83	6.09
Clean reads (G)	6.13	7.47	6.44	7.14	5.81	7.10	5.76	6.04
Mean coverage	294×	4334×	1695×	1626×	1197×	1549×	352×	2590×
GenBank numbers	MT409612	MT409613	MT409614	MT409615	MT409616	MT409617	MT409618	MT409619
Plastome size (bp)	147,808	155,623	147,797	147,752	148,196	146,443	148,107	147,462
LSC (bp)	91,907	85,066	91,846	91,782	92,305	92,598	92,214	93,363
IRs (bp)	19,199	26,908	19,162	19,205	19,158	18,166	19,156	18,254
SSC (bp)	17,503	16,741	17,627	17,560	17,575	17,513	17,581	17,591
Total GC content (%)	37.5%	37.6%	37.3%	37.4%	37.5%	37.5%	37.5%	37.6%
LSC (%)	36.0%	35.7%	35.9%	35.9%	35.9%	35.9%	36.0%	36.0%
IR (%)	44.1%	42.5%	44.1%	44.0%	44.1%	44.8%	44.1%	44.8%
SSC (%)	31.0%	31.0%	30.9%	30.9%	31.0%	31.2%	31.0%	31.1%
Total gene numbers	129	133	129	129	129	129	129	129
Protein-coding	85(5)	88 (8)	85(5)	85(5)	85(5)	85(5)	85(5)	85(5)
tRNA	36 (6)	37 (7)	36 (6)	36 (6)	36 (6)	36 (6)	36 (6)	36 (6)
rRNA	8 (4)	8 (4)	8 (4)	8 (4)	8 (4)	8 (4)	8 (4)	8 (4)

### Codon usage and RNA editing sites

**Fig. 2:**
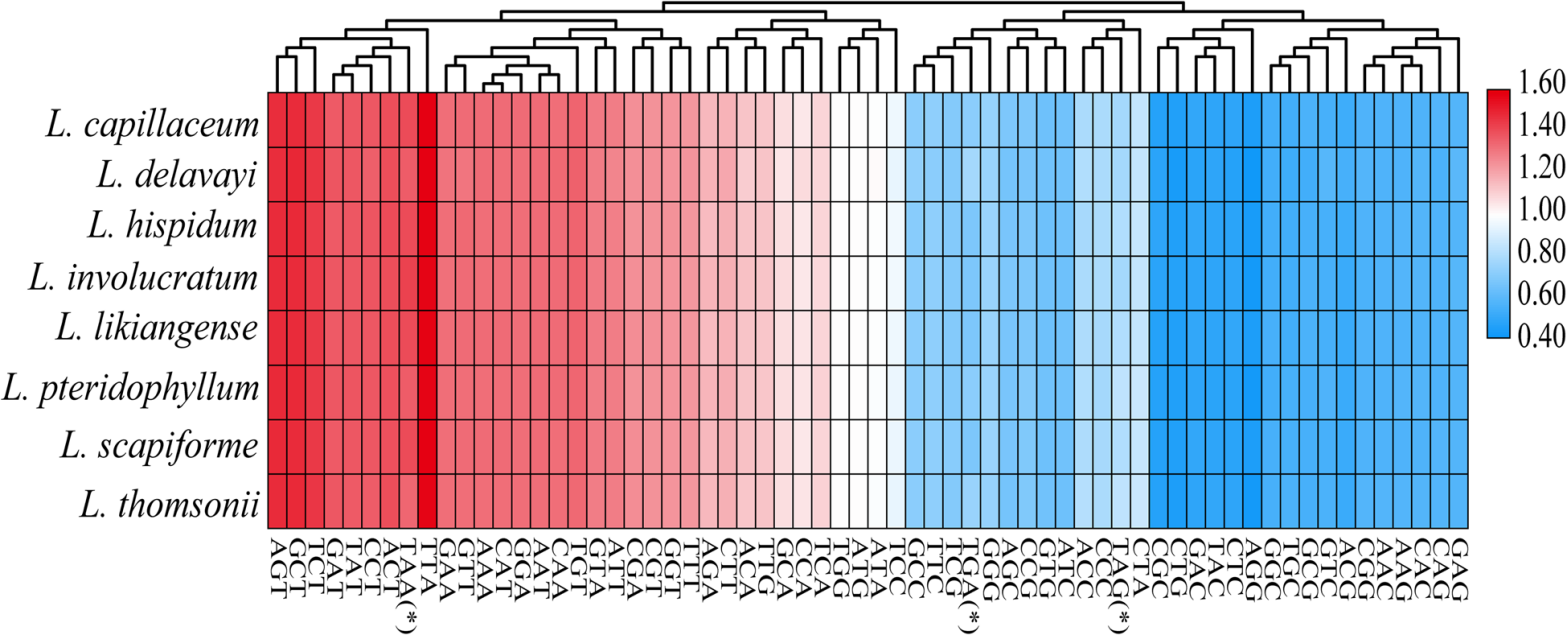
The RSCU values of all merged protein-coding genes for eight *Ligusticum* plastomes. Color key: the red values indicate higher RSCU values and the blue values indicate lower RSCU values

The total sequence sizes of the protein-coding genes for codon analysis were 67,905–68,268 bp in the eight *Ligusticum* plastomes. These protein sequences encoded 22,635–22,756 codons, which are summarized in Additional file 3: Table S2. Leu was encoded by the highest number of codons (2382-2419), whereas Cys was the least (232–241). The RSCU values of all codons in the form of a heatmap are shown in Fig. 2. The red values indicate higher RSCU values and the blue values indicate lower RSCU values. The heatmap showed that about half of the codons were used more frequently. Specifically, 30 codons were used frequently with RSCU > 1, and all biased codons ended with a purine (A/T) except TTG (Fig. 2, Additional file 3: Table S2). The mean values of GC content of the first, second, and third codon positions were 46.0, 38.3, and 29.7%, respectively (Additional file 4: Table S3). This GC content also implied that the plastome of *Ligusticum* has a strong bias towards A/T at the third codon position. The usage of two codons (ATG and TGG) had no bias (RSCU = 1) (Additional file 3: Table S2).

Additionally, potential RNA editing sites were identified for 35 genes of the eight *Ligusticum* plastomes. A total of 469 RNA editing sites were identified, in which the number of editing sites ranged from 55 (*L. delavayi*) to 67 (*L. likiangense*) (Additional file 5: Table S4). The *ndhB* gene had the highest number of RNA editing sites (10) in all of the eight *Ligusticum* plastomes, whereas the *rps8* gene also had 10 RNA editing sites in *L. likiangense*. All of the identified RNA editing sites were Cytosine to Uracil (C-U) conversion and most of them were situated in the second codon position (40–51), followed by the first codon position (12–16), but no sites situated in the third codon position (Additional file 1: Figure S1). The amino acid conversion Serine to Leucine (S-L) occurred most frequently. Furthermore, a mass of RNA editing sites (420) caused amino acid changes for hydrophobic products, such as Leucine (L; 219), Phenylalanine (F; 63), Isoleucine (I; 58), Tyrosine (Y; 25), Methionine (M; 21), Tryptophan (W; 17), and Valine (V; 17) (Additional file 5: Table S4).

### Repeat element analysis

**Fig. 3:**
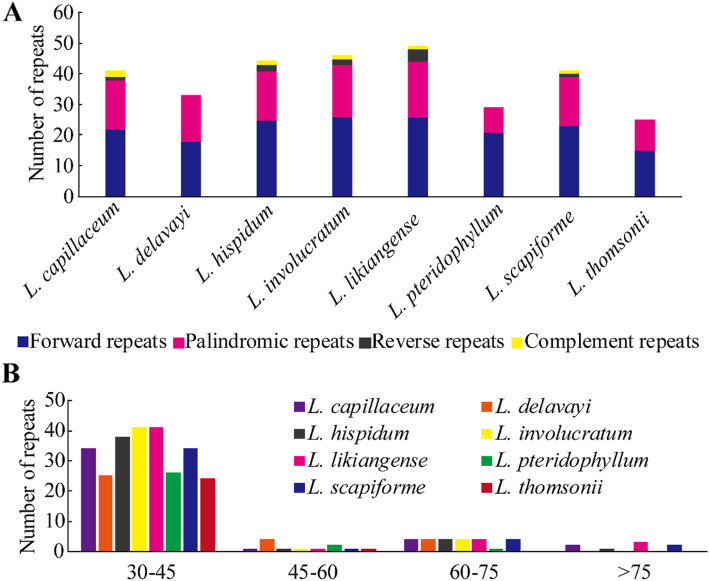
Analysis of repeat sequences in the eight *Ligusticum* plastomes. **a** Total numbers of four repeat types. **b** Number of repeats divided by length

Forward, palindromic, reverse, and complementary repeats were detected in the eight *Ligusticum* plastomes. In all, we detected 308 repeats with 30–82 bp long (Additional file 6: Table S5). The number of forward repeats (176) was higher than that of palindromic repeats (116), reverse repeats (10), and palindromic repeats (6). *L. likiangense* contained the most repeats (49), while *L. thomsonii* contained the least (25) (Fig. 3). According to the length, we artificially divided the repeats into four categories: 30–45 bp, 45–60 bp, 60–75 bp, and > 75 bp (Fig. 3). Among them, most of the repeats (85%) were 30–45 bp long. The majority of the repeats were located in intergenic or intron regions (70.5%), and a minority were located in gene regions (29.5%). 603 simple sequence repeats (SSRs) were detected, but the number of SSRs differed among eight *Ligusticum* species (Additional file 1: Figure S2, Additional file 7: Table S6). *L. scapiforme* contained the most SSRs (82), while *L. delavayi* and *L. hispidum* contained the least (68). The most abundant were mononucleotide repeats (54.9%), followed by dinucleotides (25.5%), tetranucleotides (12.3%), trinucleotides (3.0%), and pentanucleotides (3.0%). Hexanucleotides are very rare across the plastomes. SSRs were distributed mainly in the LSC (68.3%), with less in the IRs (16.6%) and SSC (15.1%) (Additional file 7: Table S6).

### Comparisons of border and sequence identity

**Fig. 4:**
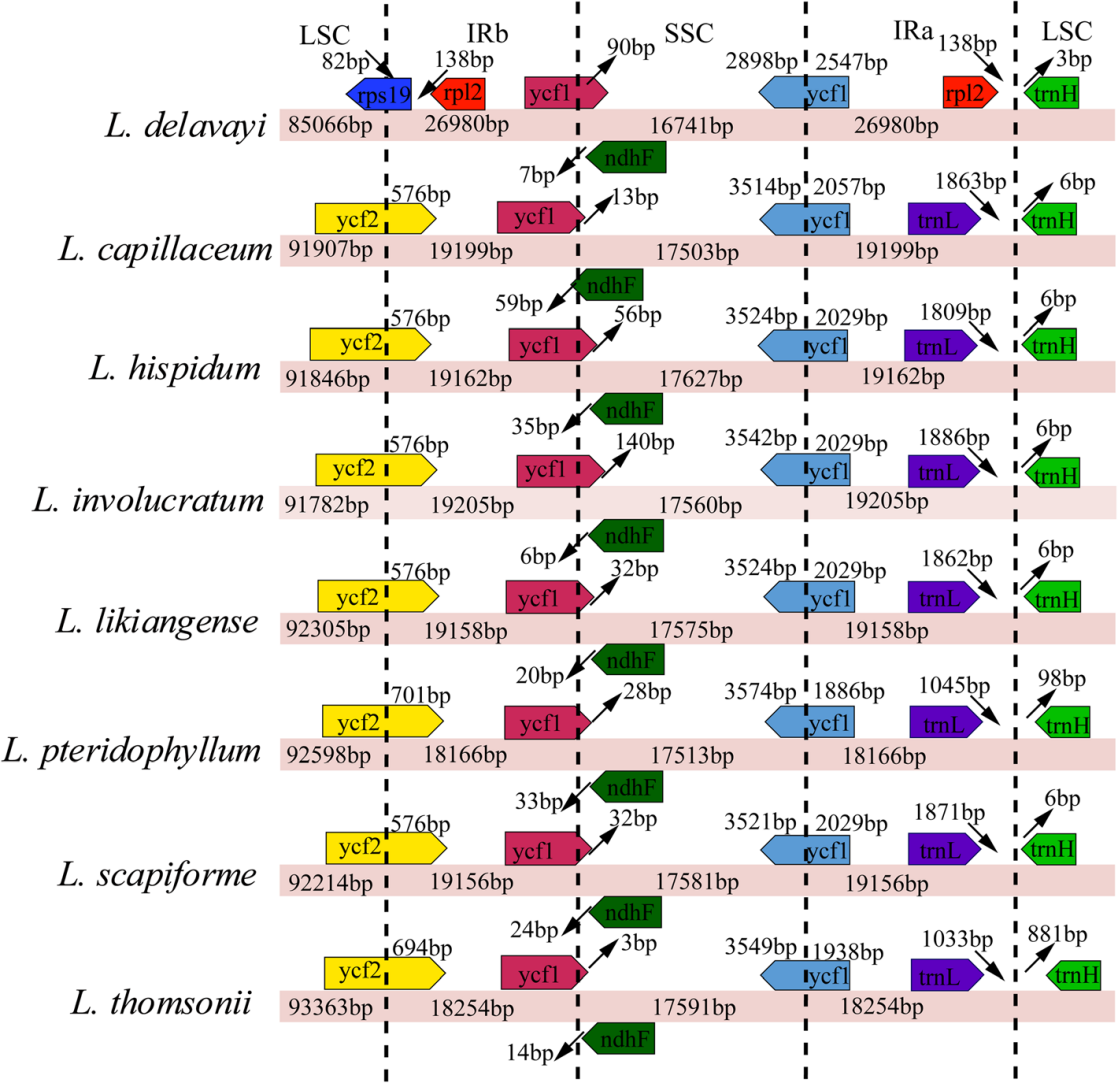
Comparison of the border regions of the eight *Ligusticum* plastomes. LSC (large single copy), SSC (small single copy), and IR (inverted repeat) regions are indicated. This figure is not to scale

The differences between inverted repeat and single-copy (IR/SC) borders among eight *Ligusticum* plastomes were examined (Fig. 4). Besides *L. delavayi*, seven other *Ligusticum* species were conserved in terms of the gene order and gene content at the IR/SC borders. For *L. delavayi*, the LSC/IRb border was *rps19*/*rpl2* genes and the IRa/LSC border was *rpl2*/*trnH* genes. For the other *Ligusticum* species, LSC/IRb borders extended 576–701 bp into the *ycf2* gene. The SSC/IRb borders extended 3–140 bp into the *ycf1* genes, where the smallest and largest extensions occurred in *L. thomsonii* (3 bp) and *L. involucratum* (140 bp). The *ndhF* gene in *L. capillaceum* overlapped with the SSC/IRb border by 59 bp. The *ycf1* genes, crossing the SSC/IRa borders, were located at the SSC and IRa regions with 3514–3574 bp and 1886–2057 bp. The *trnL* and *trnH* genes were 1033–1871 bp and 6–881 bp away from the IRa/LSC borders.

**Fig. 5:**
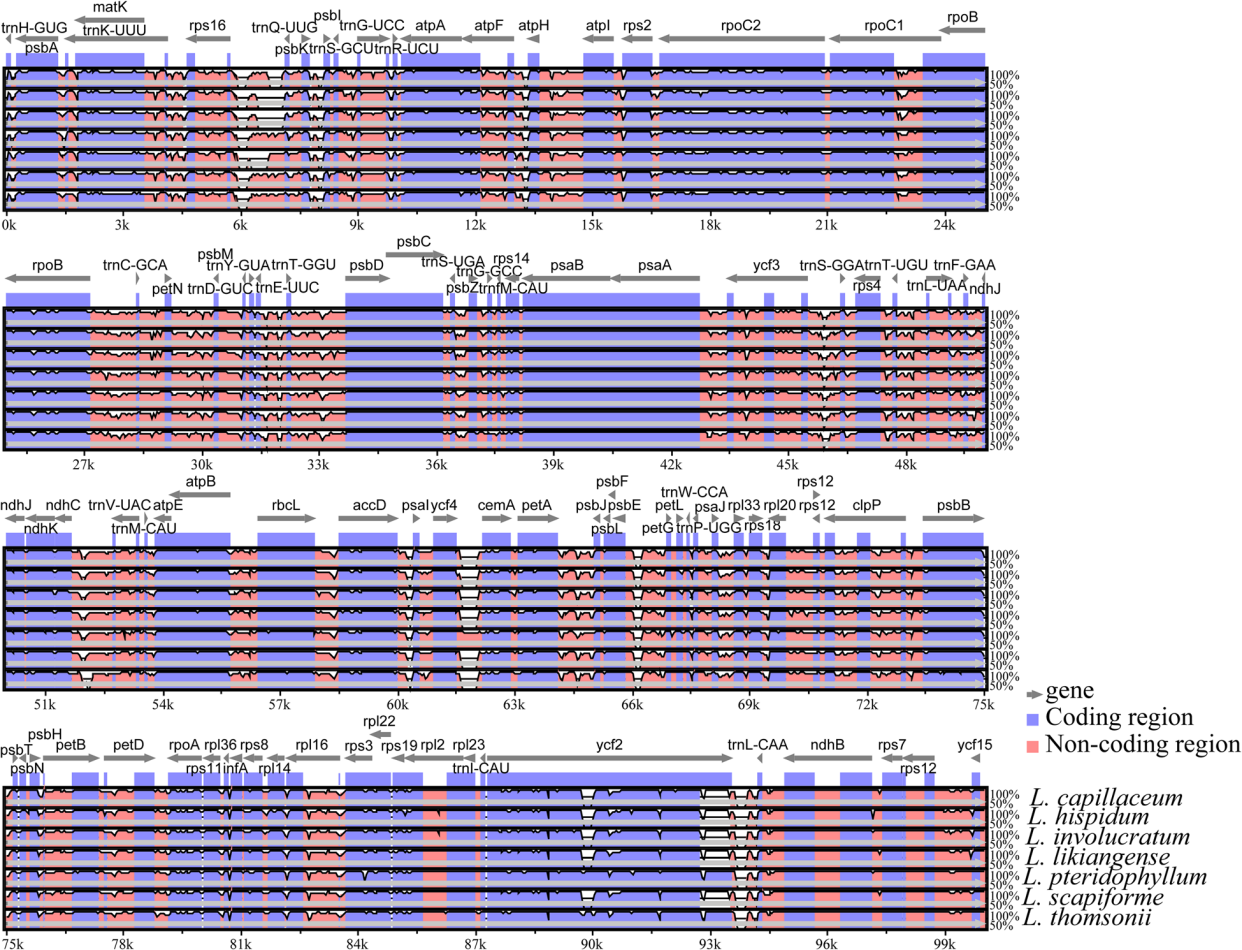
VISTA-based sequence identity plot of the eight *Ligusticum* plastomes using *L. delavayi* as a reference. The vertical scale represents the percentage of identity ranging from 50 to 100%. Coding and non-coding regions are marked in purple and pink, respectively

**Fig. 6:**
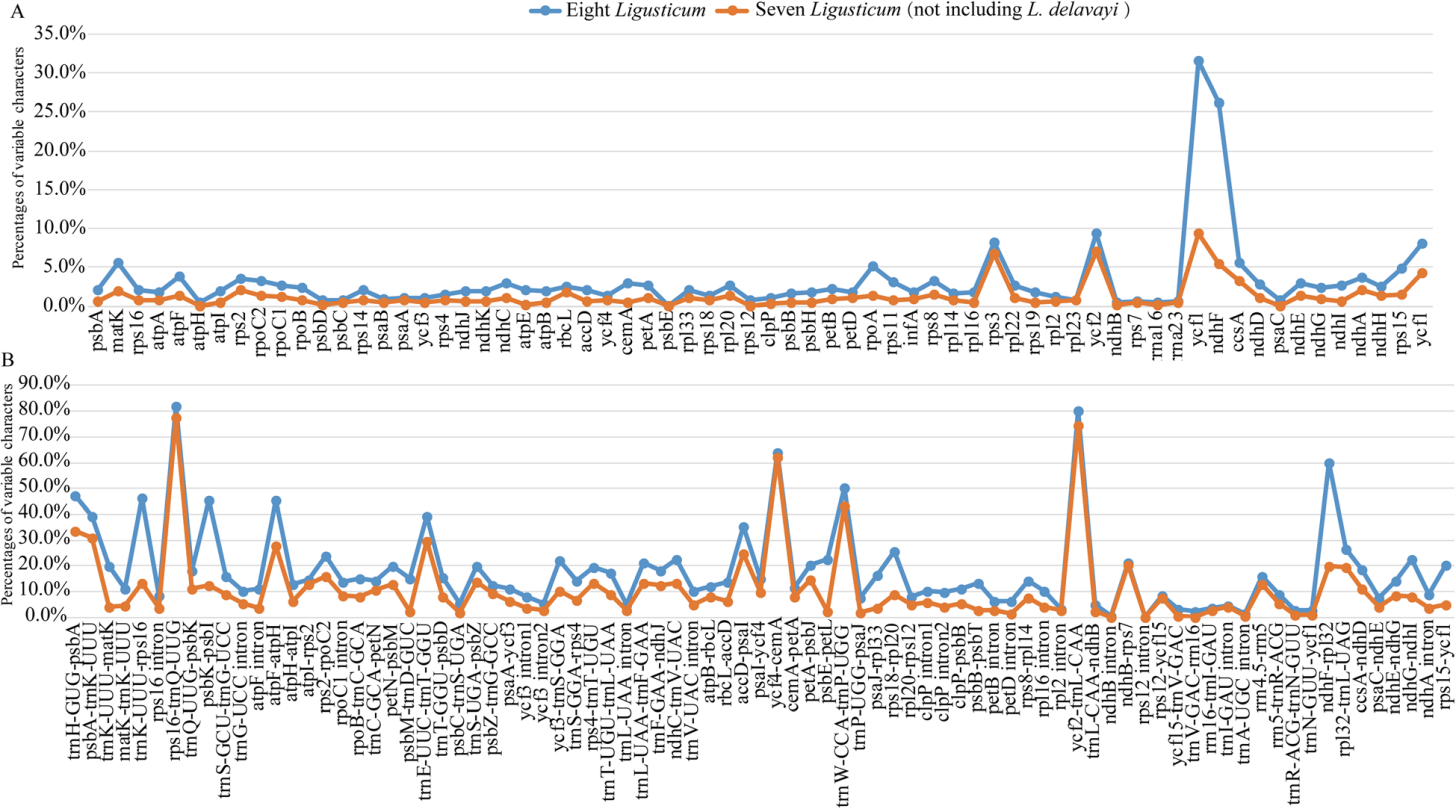
Percentage of variable characters in aligned *Ligusticum* plastomes. **a** Coding region. **b** Non-coding region. The blue and orange lines show the eight *Ligusticum* and seven *Ligusticum* (not including *L. delavayi*). These regions are oriented according to their locations in the plastome

The mVISTA program was used to conduct a sequence identity analysis using *L. delavayi* as a reference. The results are revealed in Fig. 5, of which coding regions showed more sequence conservation than non-coding regions. 766 SNPs and 351 Indels were detected among the eight *Ligusticum* plastomes (Additional file 8: Table S7). The majority of SNPs and Indels (786) were from non-coding regions, while a minority (331) were from coding regions. We also identified the average percentage of variation for 149 regions (66 coding regions, 64 intergenic spacers, and 19 introns) (Fig. 6, Additional file 9: Table S8). Among these regions, the average percentage of variation for non-coding regions (18.5%) was higher than that (3.2%) for coding regions (Additional file 9: Table S8). Twelve non-coding regions exhibited high variation: *trnH-GUG/psbA*, *psbA/trnK-UUU*, *trnK-UUU/rps16*, *rps16/trnQ-UUG*, *psbK/psbI*, *atpF/atpH*, *trnE-UUC/trnT-GGU*, *accD/psaI*, *ycf4/cemA*, *trnW-CCA/trnP-UGG*, *ycf2/trnL-CAA*, and *ndhF/rpl32* (the percentage of variation > 30%). Eight coding regions exhibited high variation: *matK*, *rps3*, *ycf2*, *ycf1* × 2, *ndhF*, *rpoA*, and *ccsA* (the percentage of variation > 5%). Seven other *Ligusticum* species showed sequence differences when *L. delavayi* was a reference in the mVISTA plot (Fig. 5). Therefore, we calculated the percentages of variable characters for coding and non-coding region of seven *Ligusticum* species (not including *L. delavayi*), as well as the genetic distance of the eight *Ligusticum* plastomes. The pairwise genetic distance ranged from 0.0010 to 0.0239 with an overall average was 0.0092 (Additional file 10: Table S9). However, the values of pairwise genetic distance between *L. delavayi* and seven other *Ligusticum* species were higher: 0.0234 (*L. capillaceum*), 0.0236 (*L. scapiforme*), 0.0234 (*L. likiangense*), 0.0237 (*L. hispidum*), 0.0239 (*L. involucratum*), 0.0221 (*L. pteridophyllum*), and 0.0232 (*L. thomsonii*). Figure 6 shows that the sequence difference of seven other *Ligusticum* species was lower without *L. delavayi*. In a word, our results demonstrated that *L. delavayi* showed a higher sequence difference than the rest seven *Ligusticum* species (Fig. 6, Additional file 10: Table S9).

### Selective pressure in plastid genes

The dN/dS ratios of the 79 common protein-coding genes were calculated to estimate selective pressures (Additional file 11: Table S10). The dN/dS ratios of the most genes in our results were less than 0.5, suggested that they were under the purifying selection. Despite this, we also detected an increase in dN/dS, indicating relaxed selection in nine genes (0.5 < dN/dS < 1.0). Unexpectedly, none but four genes were significant (*P* < 0.05) after the likelihood ratio test (LRT). Therefore, the analyses presented here demonstrated that four genes were under relaxed selection, namely *ccsA*, *rpoA*, *ycf1*, and *ycf2* (Additional file 11: Table S10). Meanwhile, only one gene with dN/dS > 1.0 (*psaJ*), but the LRT was not significant (*P* > 0.05).

### Phylogenetic relationships

**Fig. 7:**
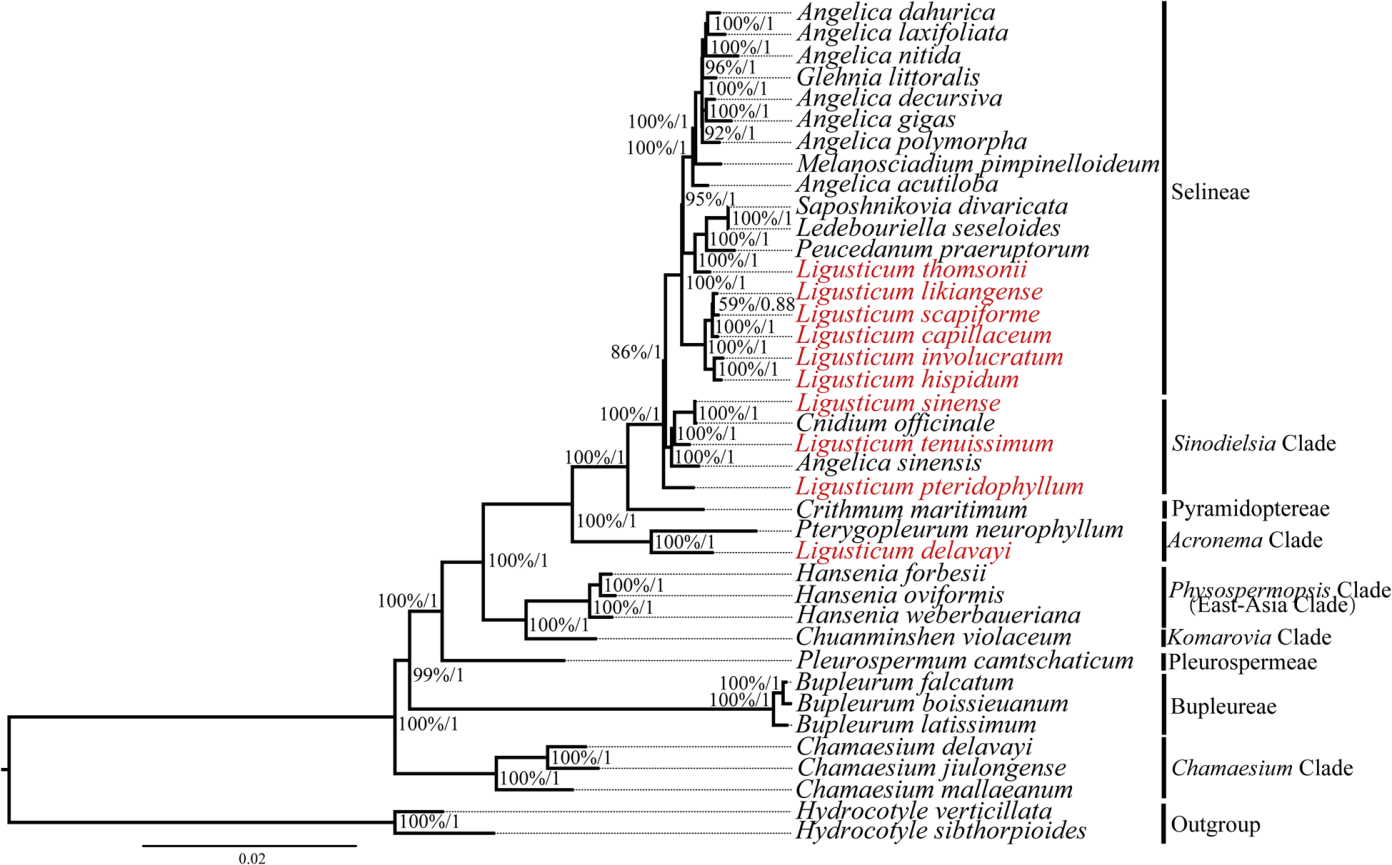
Phylogenetic tree reconstruction of the 39 species inferred from Maximum likelihood (ML) and Bayesian inference (BI) analyses based on the complete plastomes. The bootstrap support values and posterior probability values are listed at each node

39 complete plastomes and 80 nuclear ITS sequences were used to carry out the phylogenetic analyses (Additional file 12: Table S11). The plastome tree and ITS tree produced incongruent tree topologies, while they all inferred the non-monophyly of *Ligusticum* species (Fig. 7, Additional file 1: Figure S3). In the plastome tree, the *L. capillaceum*, *L. scapiforme*, *L. likiangense*, *L. hispidum*, *L. involucratum*, and *L. thomsonii* belonged to Selineae. However, five other *Ligusticum* species formed a clade, *L. thomsonii* clustered with *S. divaricata*, *L. seseloides*, and *P. praeruptorum*. *L. tenuissimum* and *L. sinense* belonged to *Sinodielsia* Clade, but they did not form a clade. *L. sinense* was more closely related to *C. officinale*, then they clustered with *L. tenuissimum*. *L. delavayi* always clustered with *P. neurophyllum*, belonged to *Acronrma* Clade. *L. pteridophyllum* belonged to *Sinodielsia* Clade [4], while it was resolved as sister to *Sinodielsia* Clade + Selineae. In the ITS tree, the *L. capillaceum*, *L. scapiforme*, *L. likiangense*, *L. hispidum*, *L. involucratum*, and *L. thomsonii* formed a clade and belonged to Selineae. *L. tenuissimum* was resolved as sister to Selineae with weak support (BS = 54%, PP = 0.8). *L. sinense* was still more closely related to *C. officinale*. The systematic position of *L. delavayi* was in line with the plastome tree. The ITS tree topologies resulting from ML and BI analysis were some different. For example, *L. pteridophyllum* clustered with *L. sinense* and *C. officinale* by BI analysis (PP = 0.57), whereas it was the parallel branch’s relationships with *L. sinense* and *C. officinale* by ML analysis (BB = 57%). In Selineae, six *Ligusticum* and seven *Angelica* falled within this tribe. Like *Ligusticum*, *Angelica* also showed polyphyly, and *M. pimpinelloideum* and *G. littoralis* embedded in it. *Chamaesium* Clade was the basal taxa of the Apioideae rather than Bupleureae. *Sinodielsia* Clade was not a monophyletic group in our phylogenetic analyses.

## Discussion

### Plastome evolution

The plastomes are highly conserved in genome structure, gene order, and gene content [19, 21, 26]. Nevertheless, genome rearrangement, gene loss (pseudogenization or deletion), differences in structure and size, expansions and contractions of IR have been confirmed to occur many times during plastome evolution [20, 27–30]. In this study, the *Ligusticum* plastomes were low conservation, exhibiting striking differences in terms of genome size (146,443–155,623 bp), gene number (129–133), and IR/SC borders. *L. delavayi* had the longest plastome length, whereas its LSC and SSC regions were shorter than that of seven other *Ligusticum* species. The differences in length reflect the extensions of IRs and the contractions of two SCs. It had four more genes (*ycf2*, *rpl23*, *rpl2*, and *trnI-CAU*) than seven other *Ligusticum* species in IRa, which was likely ascribed to the gene duplication resulting from the extensions of IRs. The varied gene number in the congeneric species has been found in other angiosperm plastomes [31, 32]. For *L. delavayi*, the IRa/LSC border was *rpl2*/*trnH* genes and the LSC/IRb border was *rps19*/*rpl2* genes, which were different from seven other *Ligusticum* species (*trnL*/*trnH* and *ycf2*). The IRa region extended into the *ycf1* gene was a common feature in plastome evolution [31, 33, 34]. The IR regions had a higher GC content of 42.5–44.8% possibly caused by the high GC content of four rRNA genes [34].

SSRs have been used widely in plant population genetics and evolutionary studies [35, 36]. The most abundant SSRs were mononucleotide in the eight *Ligusticum* plastomes, followed by dinucleotide, tetranucleotide, trinucleotide, pentanucleotide, and hexanucleotide repeats. This phenomenon has been reported in *Primula* [31] and *Allium* [37]. The most probable explanation for the largest amount of SSRs in the LSC is that LSC is longer than SSC and IRs. The majority of the SSRs contained A/T motifs, causing the AT richness of the overall plastome [34]. The cpSSRs reported here are informative sources for developing molecular markers for genetic diversity studies of *Ligusticum* species.

Codons with a higher AT content are usually used in plastomes, and the trend is more striking for A/T use in the third codon positions [38]. The bias also showed in the eight *Ligusticum* plastomes. Leucine was encoded by the highest number of codons, and the order of codon preference was TTA > CTT > TTG > CTA > CTC > CTG, which following most Geraniaceae species [20]. RNA editing is an important process to regulate the gene expression of posttranscriptional in plant organelles [39]. The events occur in all major lineages of land plants, except *Marchantia polymorpha* and some green algae [40]. RNA editing can correct DNA mutations at the RNA level, thus recovers conserved amino acid residues to maintain functions of encoded proteins [41–43]. Most of the editing sites occurred at the second codon position and no sites occurred at the third codon position, the distribution pattern was also found in *Forsythia suspensa* [44]. Like many other plants, the *ndhB* gene had the most RNA editing sites [40, 45], which suggests that *ndhB* gene is critical in regulating plant physiological and biochemical processes.

Large repeat sequences are considered to be the major cause to promote plastome rearrangement and sequence divergence [46–48]. Among the identified 308 repeats, short repeat with 30–45 bp (85.4%) was the most, which was consistent with many unrearranged plastomes [49, 50]. Moreover, non-coding regions distributed more SNPs and Indels and had a higher average percentage of variation than that of coding regions. Consequently, our study showed that non-coding regions were less conservative than coding regions. The distribution of repeats is correlated with mutational events, and repeats may play a role in inducing mutations [30, 51, 52]. Our results also indicated that the distribution of repeats was relevant to mutational events, for repeats located predominantly in highly variable non-coding regions (70.5%) instead of coding regions (29.5%). DNA barcodes are defined as the short DNA sequences with adequate variations to identify species in the given taxonomic group [53]. Eight coding regions and 12 non-coding regions with the highest percentage of variation have been described. Thereinto, several regions have been ascertained in other angiosperms, such as *matK*, *ndhF*, *rps3*, *ycf2*, *ycf1* × 2, *rpoA*, *trnH-GUG/psbA*, *ndhF/rpl32*, *trnK-UUU/rps16*, *psbK/psbI*, *ycf4/cemA*, *accD/psaI*, *ycf2/trnL-CAA*, *rps16/trnQ-UUG*, and *trnE-UUC/trnT-GGU* [22, 49, 50, 54–56]. For the herbal medicinal genus *Ligusticum*, these regions could serve as candidate DNA barcodes for species authentication to assure medicinal quality.

We estimated the selective pressures of 79 common protein-coding genes in *Ligusticum* plastomes. Most of them were under purifying selection, which reflected the typically evolutionary conservation of plastid genes in plants [57, 58]. Four genes (*ycf1, ycf2*, *ccsA*, and *rpoA*) were under relaxed selection. The *ycf1* and *ycf2* genes, the largest and the second-largest genes in the plastome, have been proved to be absent or pseudogenized in many prior works [21, 59]. Relaxed selection on the two genes also has been observed in *Corallorhiza striata*, *Lennoa madreporoides,* and *Pholisma arenarium* [60, 61]. The genes *rpoA* and *ccsA* encode an α subunit of RNA polymerase and a protein required for heme attachment to C-type cytochrome, respectively [62, 63]. They usually present in land plants, whereas they are absent from the plastome of *Physcomitrella patens* [63]. Nevertheless, the plastomes of parasitic plants generally are the best model systems to study the effect of relaxed selection on photosynthetic function [64, 65]. Indeed, some parasitic plants harbor drastically reduced plastome size and gene content resulting from the relaxed selection on photosynthesis-related genes [60, 64, 66]. Therefore, further studies are necessary to investigate the important role of relaxed selection in *Ligusticum* plastid genes. Overall, these findings shed new lights on the plastid genes of *Ligusticum* species.

### Phylogenetic relationships

Until now, molecular phylogenetic studies based on a few molecular markers do not support the monophyly of *Ligusticum* species [4, 8–14]. Here, we performed phylogenetic analyses for *Ligusticum* using complete plastomes and ITS sequences. Unfortunately, we still failed to recognize *Ligusticum* as a monophyletic group. The plastome tree and ITS tree produced incongruent tree topologies. *L. capillaceum*, *L. scapiforme*, *L. likiangense*, *L. hispidum*, and *L. involucratum* share some similar morphological characteristics (e.g., bracteole pinnate and stem bases clothed in fibrous remnant sheaths), and they formed a clade (BS = 100%, PP = 1) in the plastome tree. However, *L. thomsonii* clustered with the above five species to form a clade in the ITS tree. *L. tenuissimum* belonged to *Sinodielsia* Clade in the plastome tree, which in accord with the results of Zhou et al. [4], whereas it was resolved as sister to Selineae with weak support in the ITS tree (BS = 54%, PP = 0.8). The incongruence between nuclear and plastome phylogenies has been commonly observed in other plant lineages [67–69]. This incongruence was likely the result of different inherited background and mutation rates of ITS and plastid DNA [70, 71]. The nuclear ITS is biparentally inherited and has a higher mutation rate, whereas the plastid DNA is maternally inherited and has a lower mutation rate [70, 71]. Moreover, the hybridization and incomplete lineage sorting (ILS) may be responsible for the inconsistent relationships between ITS- and plastome-based phylogenies [72, 73]. *L. sinense* was more closely related to *C. officinale*, which can be possibly explained well using the cross-hybridization of genomes [74]. *L. pteridophyllum* clustered with *L. sinense* and *C. officinale* in ITS tree by BI analysis, which was consistent with the results of Zhou et al. [4]. These together suggested that *Ligusticum* species may have experienced a complex evolutionary history. The polyphyly of *Angelica*, as well as *Glehnia* and *Melanosciadium* embedded in it, have been documented by earlier studies [12, 14, 75]. *Sinodielsia* Clade was not recovered as a monophyletic that has been observed in other work [14]. *Chamaesium* Clade was the basal taxa of the Apioideae as a recent study based on 3351 single-copy genes [76].

The plastome tree obtained moderate-to-high support, conversely the ITS tree obtained lower support and more parallel branches. Therefore, our results highlight the advantage of plastome with mass informative sites in resolving phylogenetic relationships. This study is also the first to support the polyphyly of *Ligusticum* based on plastomes. Further studies that include greater taxon sampling are necessary to confirm the polyphyletic position of the *Ligusticum*. Moreover, combined with the previous studies [4], we considered that the current taxonomy system of *Ligusticum* needs to be improved and revised. In a word, our study provided useful information for future phylogeny, taxonomy, and evolutionary history studies of the *Ligusticum* species.

## Methods

### Taxa sampling and DNA extraction

Fresh green leaves from adult plants of eight species were sampled from the field, and then immediately dried with silica gel for the next step. Permission is not required to sample these plants because they are not key protected plants. Total genomic DNA was extracted from silica-dried leaves with a modified CTAB protocol [77]. The formal identification of the plant material was undertaken by Xingjin He (Sichuan University). Voucher specimens were deposited at the herbarium of Sichuan University (Chengdu, China) (Additional file 13: Table S12). For ITS analyses, we newly sequenced 17 ITS have been submitted into NCBI (accession numbers: MT974009-MT974025) (Additional file 12: Table S11).

### Genome sequencing, assembly, and annotation

The raw reads of the eight newly sequenced species were generated from an Illumina HiSeq X Ten platform (paired-end, 150 bp) at Novogene (Tianjin, China). Quality control of the raw reads was performed using fastP version v0.15.0 (−n 10 and -q 15) [78], yielding at least 5GB clean reads for each species. Then clean reads were used to perform a de novo assembly by NOVOPlasty v2.6.2 [79] with the default parameters. The seed sequence is the *rbcL* gene from the reference genome sequence of *L. tenuissimum* (NC_029394). The program DOGMA [80] was used to annotate the genes of the eight plastomes, and adjusted manually in Geneious v9.0.2 (Biomatters Ltd., Auckland, New Zealand) based on comparisons with its congeneric species. All of the eight newly generated complete plastomes were available in NCBI (accession numbers: MT409612-MT409619) (Table 1). The circle plastome map was drawn using the online program OrganellarGenomeDRAW (OGDRAW) [81].

### Codons, RNA editing sites, and repeat sequences

The protein-coding genes were extracted from the eight *Ligusticum* plastomes for codon analysis. All overlapping genes were removed, and the final dataset included 80 protein-coding genes for each species. Codon usage and relative synonymous codon usage (RSCU) [82] values were calculated using the CodonW v1.4.2 program [83]. The heatmap from all RSCU of the eight plastomes was produced using TBtools [84]. The base compositions for protein-coding genes were calculated by MEGA6 [85]. The online program Predictive RNA Editor for Plants suite [86] with a cutoff value of 0.8 was used to predict the potential RNA editing sites.

The online REPuter program [87] was used to identify repeat sequences, including forward, palindromic, reverse, and complementary repeats. According to the following parameters: (1) a repeat size of more than 30 bp; (2) more than 90% sequence identity between the two repeats; and (3) Hamming distance = 3. All overlapping repeat sequences were removed. The Perl script MISA (http://pgrc.ipk-gatersleben.de/misa/) was used to exploit simple sequence repeats (SSRs). The minimum number of SSRs was set to 10, 5, 4, 3, 3, and 3, for mono-, di-, tri-, tetra-, penta-, and hexanucleotides, respectively.

### Sequence divergence

The whole-genome alignment of the eight *Ligusticum* plastomes was generated and visualized using the mVISTA [88] using *L. dilavayi* as a reference. Eight *Ligusticum* plastomes were aligned in Geneious v9.0.2 (Biomatters Ltd., Auckland, New Zealand) with MAFFT v7.221 [89], subsequently, Indels and SNPs were counted and positioned using the “Find Variations/SNPs”. The percentages of variable characters for coding and non-coding regions were calculated based on the method of Zhang et al. [90]. The genetic distance of the eight *Ligusticum* plastomes was calculated using MEGA6 [85].

### Selective pressure analysis

Selective pressures were analyzed for common 79 protein-coding genes among ten *Ligusticum* species (including 2 published plastomes). The ratio (ω) of non-synonymous to synonymous nucleotide substitution rates (dN/dS) was calculated using the Codeml program in PAML4.9 with the site-specific model (seqtype = 1, model = 0, NSsites = 0, 1, 2, 3, 7, 8) [91, 92]. The codon frequencies were determined by the F3 × 4 model. We compared three sets: M0 vs M3, M1 vs M2, and M7 vs M8 to detect selected sites. The likelihood ratio test (LRT) was used to confirm the quality of the three sets. Bayes Empirical Bayes (BEB) analysis was used to statistically identify selected sites with posterior probabilities ≥95%. We classified genes as evolving under positive selection (dN/dS > 1.0), relaxed selection (0.5 < dN/dS < 1.0), and purifying selection (dN/dS < 0.5) [61, 93].

### Phylogenetic analysis

Earlier molecular systematic studies identified five clades within *Ligusticum*, including *Acronema* Clade, *Conioselinum chinense* Clade, Pyramidoptereae, Selineae, and *Sinodielsia* Clade [12]. More recently, the genus *Ligusticum* has been divided into six clades, and East-Asia (*Physospermopsis*) Clade was added [4]. Here, we used 39 complete plastomes and 80 nuclear ITS sequences to infer the phylogenetic relationships of *Ligusticum*. Sequence alignment was achieved using the MAFFT v7.221 [89]. The aligned sequence was then manually examined and corrected. Maximum likelihood (ML) and Bayesian inference (BI) methods were used to infer phylogenetic relationships. RAxML v8.2.8 [94] was used to perform the ML analysis with 1000 replicates and GTRGAMMA model as suggested (see RAxML manual). MrBayes v3.2.7 [95] was used to perform the Bayesian inference with the best substitution model was determined by Modeltest v3.7 [96]. The selected models for complete plastomes and ITS sequences in BI analyses were TVM + I + G and GTR + I + G, respectively. Markov chain Monte Carlo (MCMC) algorithm was run for two million generations, with one tree sampled every 100 generations. The MCMC convergence was determined by calculating the average standard deviation of split frequencies (ASDSF), which fell below 0.01. The first 25% of the trees were discarded as burn-in and the consensus tree generated using the remaining trees. The ITS trees were visualized and edited using Interactive Tree of Life (iTOL) [97]: nodes under 50% bootstrap support were collapsed.

## Conclusions

In this study, we determined the complete plastome sequences of eight *Ligusticum* species using a de novo assembly approach. Through a comprehensive comparative analysis, we observed that compared with the other seven species, *L. delavayi* exhibited striking differences in genome size, gene number, IR/SC borders, and sequence identity. We performed the phylogenetic analyses for *Ligusticum* using 39 complete plastomes and 80 nuclear ITS sequences and found that *Ligusticum* was not monophyletic as presented in previous studies. The hybridization and incomplete lineage sorting may be responsible for the inconsistent relationships between ITS- and plastome-based phylogenies. The phylogenetic analyses highlighted the advantage of using plastome with mass informative sites in resolving phylogenetic relationships. Our study enriches the data on the plastomes of *Ligusticum* and serves as a reference for subsequent phylogenomics studies of this genus.

## Supplementary information

**Additional file 1: Figure S1.** Number of RNA editing sites in the eight *Ligusticum* plastomes. **Figure S2.** Analysis of simple sequence repeats (SSRs) in the eight *Ligusticum* plastomes. **Figure S3.** Phylogenetic tree reconstruction of the 39 species inferred from Maximum likelihood (ML) and Bayesian inference (BI) analyses based on nuclear internal transcribed spacer (ITS) sequences. The bootstrap support values and posterior probability values are listed at each node.

**Additional file 2: Table S1.** List of genes present in the eight *Ligusticum* plastomes.

**Additional file 3: Table S2.** Codon usage and relative synonymous codon usage (RSCU) values of protein-coding genes of the eight *Ligusticum* plastomes.

**Additional file 4: Table S3.** Base compositions of protein-coding genes for the eight *Ligusticum* plastomes.

**Additional file 5: Table S4.** RNA editing sites analyses of the eight *Ligusticum* plastomes.

**Additional file 6: Table S5.** The repeat sequences distribution in the eight *Ligusticum* plastomes.

**Additional file 7: Table S6.** Simple sequence repeats (SSRs) distribution in the eight *Ligusticum* plastomes.

**Additional file 8: Table S7.** The Indel and SNP in the eight *Ligusticum* plastomes.

**Additional file 9: Table S8.** Percentages of variable characters in coding and non-coding regions.

**Additional file 10: Table S9.** Genetic distance of the eight *Ligusticum* plastomes.

**Additional file 11: Table S10.** Results of selective pressure analysis in Paml with the site-specific model.

**Additional file 12: Table S11.** List of species and their accession numbers in GenBank included in the phylogenetic analysis.

**Additional file 13: Table S12.** Collection locality and voucher information are provided for eight sequenced plastomes.

## Data Availability

Eight annotated plastomes and newly sequenced 17 ITS have been submitted into NCBI (https://www.ncbi.nlm.nih.gov) with accession numbers: MT409612-MT409619 and MT974009-MT974025, respectively.

## Abbreviations

ASDF: Average standard deviation of split frequencies
BEB: Bayes empirical bayes
BI: Bayesian inference
bp: Base pair
BS: Branch support
CDS: Protein-coding sequences
CTAB: Cetyl trimethylammonium bromide
ILS: Incomplete lineage sorting
IR: Inverted repeat
ITS: Internal transcribed spacer
LRT: Likelihood ratio test
LSC: Large single copy
MCMC: Markov chain Monte Carlo
ML: Maximum Likelihood
PP: Posterior probability
rRNA: Ribosomal RNA
RSCU: Relative synonymous codon usage
SSC: Small single copy
SSR: Simple sequence repeat
tRNA: Transfer RNA
