# Supplementary material for: Plastomes of eight Ligusticum species: characterization, genome evolution, and phylogenetic relationships
Source: BMC Plant Biol. 2020 Nov 13;20:519. doi: 10.1186/s12870-020-02696-7 (PMC7663912; doi:10.1186/s12870-020-02696-7)
Supplement: Supplementary file 1 — Additional file 1: Figure S1. Number of RNA editing sites in the eight Ligusticum plastomes. Figure S2. Analysis of simple sequence repeats (SSRs) in the eight Ligusticum plastomes. Figure S3. Phylogenetic tree reconstruction of the 39 species inferred from Maximum likelihood (ML) and Bayesian inference (BI) analyses based on nuclear internal transcribed spacer (ITS) sequences. The bootstrap support values and posterior probability values are listed at each node. [file 12870_2020_2696_MOESM1_ESM.pdf]

Additional file 1:

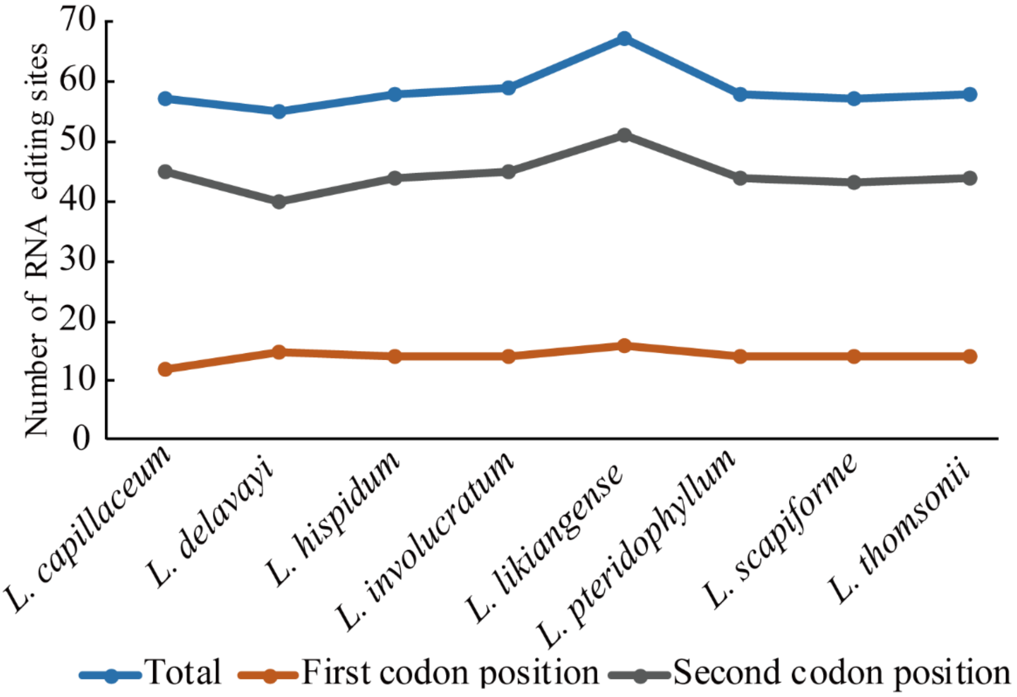

Figure S1. Number of RNA editing sites in the eight *Ligusticum* plastomes.

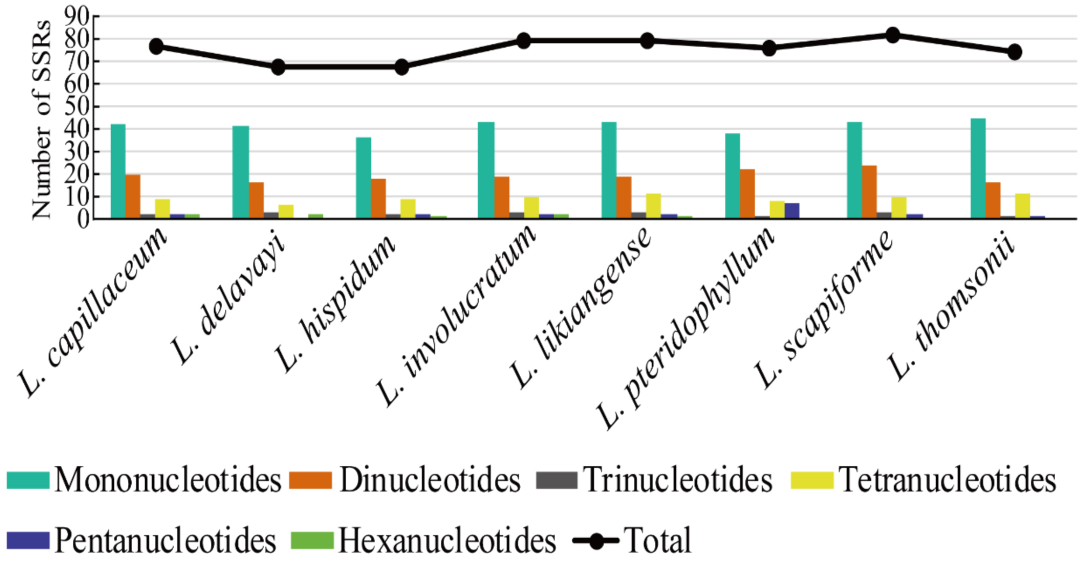

Figure S2. Analysis of simple sequence repeats (SSRs) in the eight *Ligusticum* plastomes.

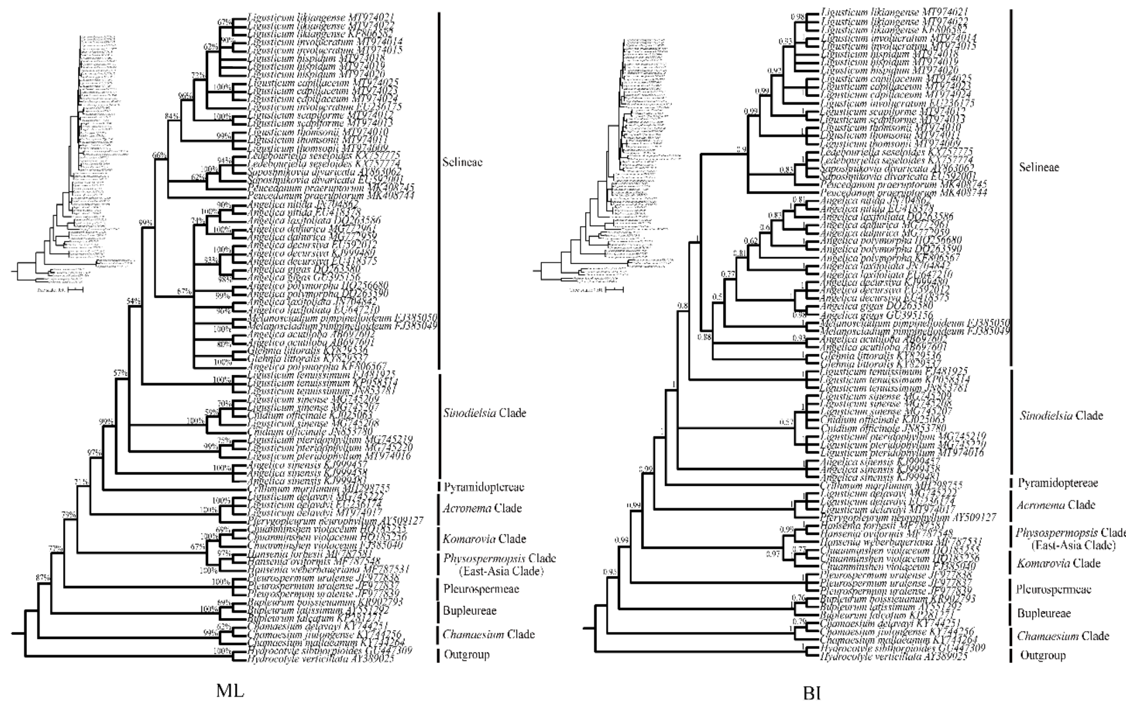

Figure S3. Phylogenetic tree reconstruction of the 39 species inferred from Maximum likelihood (ML) and Bayesian inference (BI) analyses based on nuclear internal transcribed spacer (ITS) sequences. The bootstrap support values and posterior probability values are listed at each node.
